# Genomic island excisions in *Bordetella petrii*

**DOI:** 10.1186/1471-2180-9-141

**Published:** 2009-07-18

**Authors:** Melanie Lechner, Karin Schmitt, Susanne Bauer, David Hot, Christine Hubans, Erwan Levillain, Camille Locht, Yves Lemoine, Roy Gross

**Affiliations:** 1 Lehrstuhl für Mikrobiologie, Biozentrum, Universität Würzburg, Am Hubland, D-97074 Würzburg, Germany; 2 TAG – Transcriptomics and Applied Genomics UMR8161, Institut Pasteur de Lille, Lille, France; 3 Genoscreen Company, Campus de l'Institut Pasteur de Lille, Lille, France; 4 Mécanismes Moléculaires de la Pathogénie Bactérienne, INSERM U 629, Institut Pasteur de Lille, Lille, France; 5 IFR 142, Molecular and Cellular Medecine, Institut Pasteur de Lille, Lille, France

## Abstract

**Background:**

Among the members of the genus *Bordetella B. petrii *is unique, since it is the only species isolated from the environment, while the pathogenic Bordetellae are obligately associated with host organisms. Another feature distinguishing *B. petrii *from the other sequenced Bordetellae is the presence of a large number of mobile genetic elements including several large genomic regions with typical characteristics of genomic islands collectively known as integrative and conjugative elements (ICEs). These elements mainly encode accessory metabolic factors enabling this bacterium to grow on a large repertoire of aromatic compounds.

**Results:**

During *in vitro *culture of *Bordetella petrii *colony variants appear frequently. We show that this variability can be attributed to the presence of a large number of metastable mobile genetic elements on its chromosome. In fact, the genome sequence of *B. petrii *revealed the presence of at least seven large genomic islands mostly encoding accessory metabolic functions involved in the degradation of aromatic compounds and detoxification of heavy metals. Four of these islands (termed GI1 to GI3 and GI6) are highly related to ICE*clc *of *Pseudomonas knackmussii *sp. strain B13. Here we present first data about the molecular characterization of these islands. We defined the exact borders of each island and we show that during standard culture of the bacteria these islands get excised from the chromosome. For all but one of these islands (GI5) we could detect circular intermediates. For the *clc*-like elements GI1 to GI3 of *B. petrii *we provide evidence that tandem insertion of these islands which all encode highly related integrases and attachment sites may also lead to incorporation of genomic DNA which originally was not part of the island and to the formation of huge composite islands. By integration of a tetracycline resistance cassette into GI3 we found this island to be rather unstable and to be lost from the bacterial population within about 100 consecutive generations. Furthermore, we show that GI3 is self transmissible and by conjugation can be transferred to *B. bronchiseptica *thus proving it to be an active integrative and conjugative element

**Conclusion:**

The results show that phenotypic variation of *B. petrii *is correlated with the presence of genomic islands. Tandem integration of related islands may contribute to island evolution by the acquisition of genes originally belonging to the bacterial core genome. In conclusion, *B. petrii *appears to be the first member of the genus in which horizontal gene transfer events have massively shaped its genome structure.

## Background

The enormous impact of horizontal gene transfer (HGT) on the evolution of bacterial species has only been recognized during the past years [1]. Among the mobile genetic elements involved in HGT genomic islands are of particular relevance since they can comprise large genomic regions encoding accessory factors required by the bacteria to thrive in specific environments. For example, many virulence related factors of pathogenic bacteria are encoded on so-called pathogenicity islands, while metabolic islands frequently encode factors required for detoxification of poisonous compounds or for the utilization of specific carbon sources such as aromatic compounds [2,3].

The genus *Bordetella *harbours several important pathogens infecting humans and various animals. While *B. pertussis *and *B. parapertussis *are etiological agents of whooping cough in man, *B. bronchiseptica *and *B. avium *can cause respiratory infections in various mammalian species and birds, respectively [4]. *B. petrii *was the first *Bordetella *species isolated from the environment, while all other *Bordetella *species so far could only be found in obligate association with host organisms [5]. Phylogenetically, *B. petrii *appears to be closely related to a common ancestor of the pathogenic Bordetellae and links the genus with other environmental bacteria of the genera *Achromobacter *and *Alcaligenes *[5,6]. *B. petrii *was repeatedly isolated from contaminated soil [7,8]. However, recently, several isolates from clinical specimens associated with bone degenerative disease or cystic fibrosis were found to be closely related to *B. petrii*, although the underlying etiology is not clear in any of the cases [9-11]. The pathogenic Bordetellae encode a multitude of virulence factors including several toxins and adhesins [4]. The evolutionary origin of these factors is unclear, since in contrast to many virulence genes of other pathogens they are not located on mobile genetic elements such as pathogenicity islands or prophages. In fact, so far only few presumptive horizontal gene transfer events are known among the pathogenic members of the genus, e.g. a 66 kb island encoding iron transport genes that presumably has been exchanged between *B. pertussis *and *B. holmesii*, a pathogenic species mainly found in immunocompromised individuals [12]. A prevalent feature in the evolution of virulence in this genus is reductive genome evolution, since strains specialized on particular host organisms such as the exclusive human pathogen *B. pertussis *have presumably evolved from a *B. bronchiseptica*-like ancestor. Specialization to a single host was accompanied by a massive genome size reduction. In agreement with this assumption, *B. pertussis *harbors numerous pseudogenes and virtually all *B. pertussis *genes have counterparts in *B. bronchiseptica *[13].

**Figure 1:**
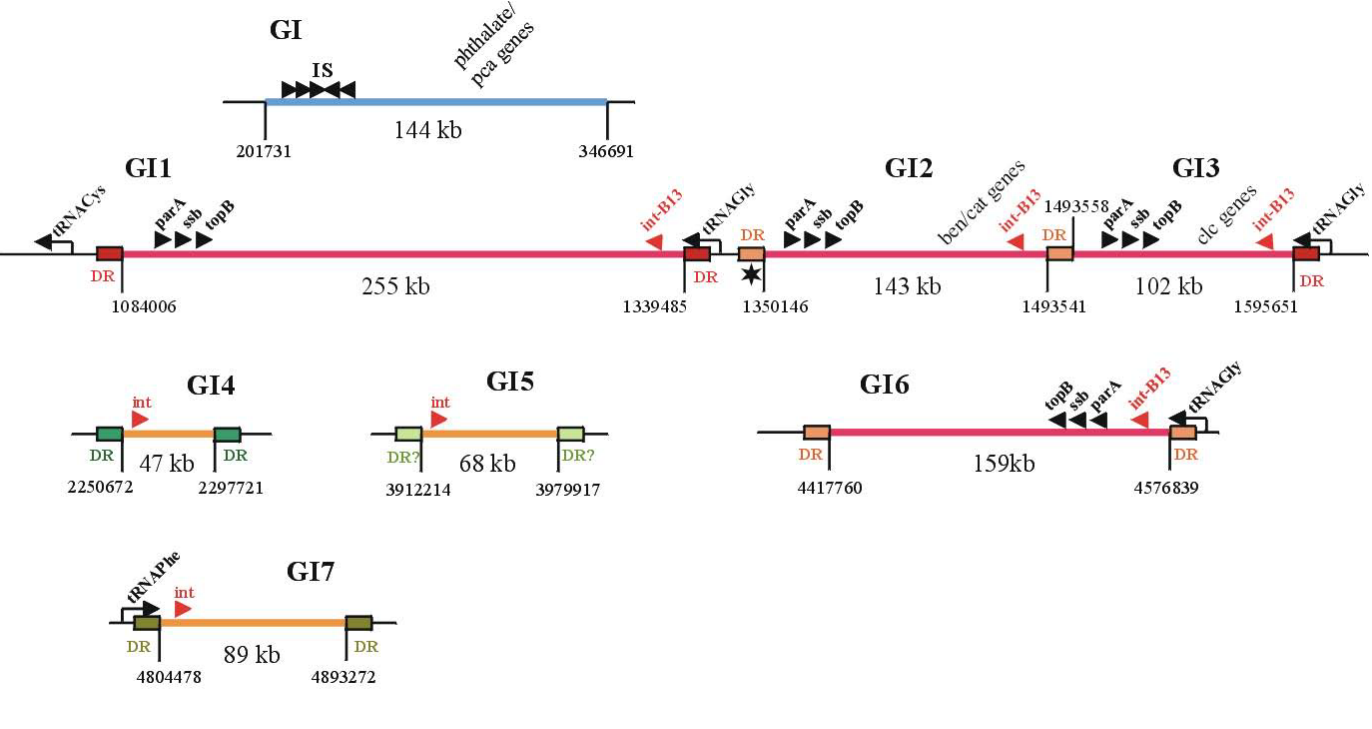
**A schematic presentation of the genomic islands described for *B. petrii *by bioinformatic analysis is shown **[14]. Direct repeats (DR) flanking the islands and their sequence position in the *B. petrii *genome are indicated. Direct repeats with identical or nearly identical DNA sequence are shown in the same colour (see also Figure 4). The approximate location of several characteristic genes such as the *parA*, *ssb *and *topB *genes found on all *clc*-like elements, integrases (*int*), or some relevant metabolic functions encoded by the islands are shown. In case tRNA genes are associated with the islands these are shown with an arrow indicating their transcriptional polarity. Finally, the approximate sizes of the predicted islands are indicated.

In contrast to *B. bronchiseptica*, *B. petrii *has a highly mosaic genome harbouring numerous mobile elements including genomic islands, prophages and insertion elements. These mobile elements comprise about 22% of the entire genome [14]. Most of the seven putative genomic islands found in *B. petrii *exhibit typical features of such islands such as a low GC content, the presence of integrase genes, conjugal transfer functions, and integration at tRNA loci (Figure 1). There are four elements (GI1–GI3, GI6) which strongly resemble the ICE*clc *of *Pseudomonas knackmussii *sp. train B13, a self transmissible element encoding factors for the degradation of chloroaromatic compounds [14-16]. The *Bordetella *islands exhibit a high similarity with the ICE*clc *in particular in a core region comprising a highly similar integrase and genes involved in conjugal transfer [14]. Like the ICE*clc *the *B. petrii *elements are characterized by the insertion into tRNA^Gly ^genes and by direct repeats formed at the insertion site [14]. The *B. petrii *islands encode factors required for degradation of a variety of aromatic compounds, or multi drug efflux pumps and iron transport functions [14].

The remaining genomic islands, GI4, GI5, and GI7, encode type IV secretion systems probably involved in conjugal transfer [14]. GI4 has very pronounced similarities with Tn*4371 *of *Ralstonia oxalatica *and other bacteria including *Achromobacter georgiopolitanum *and encodes metabolic functions involved in the degradation of aromatic compounds [17]. GI5 and GI7 encode a phage P4 related integrase and genes involved in metabolism of aromatic compounds or in detoxification of heavy metals. Finally, there is a region on the *B. petrii *genome (termed GI in [14]) which is characterized by a low GC content, but does not have other characteristic features of a genomic island thus possibly being a remnant of a former mobile element. GI encodes metabolic functions for the degradation of phthalate and protocatechuate [14].

In the present study we characterize these putative genomic islands and show that most of them are in fact active, at least in terms of excision from the chromosome. We show that these elements are responsible for the genomic instability of *B. petrii *observed during long term growth *in vitro*.

## Results and discussion

### Long term survival of *B. petrii *in river water and appearance of phenotypic variants

*B. petrii *was the first *Bordetella *species isolated from the environment, i.e. from a river sediment. The analysis of its survival capacity in river water revealed a high survival rate and nearly no decay in viability during a period of 38 weeks, while under the same experimental conditions viability of a *B. bronchiseptica *strain declined rapidly and no viable bacteria could be detected in the water samples after about three weeks (data not shown). The short survival time of *B. bronchiseptica *is somewhat surprising, since in a previous study it was shown to persist for more than 20 weeks in lake water [18]. A possible explanation for this may be that different *B. bronchiseptica *strains were used in these studies. However, the direct comparison of *B. bronchiseptica *and *B. petrii *demonstrates that *B. petrii *has a much more pronounced capacity to survive in river water for a prolonged time period which is in agreement with its original isolation from river sediment. Interestingly, after about 20 days of the survival experiment stable phenotypic variants of *B. petrii *with differing colony morphology regarding colour and colony size appeared when the bacteria were plated on LB agar plates (data not shown). In this study, three of these variants (named f, g, k) were further characterized. All of these variants showed virtually identical growth characteristics at 37°C in liquid LB medium, while two of them (f, k) showed a markedly impaired growth capacity at 15°C as compared to the wild type strain and to variant g (data not shown).

### Genome rearrangements involving the genomic islands of *B. petrii*

**Figure 2:**
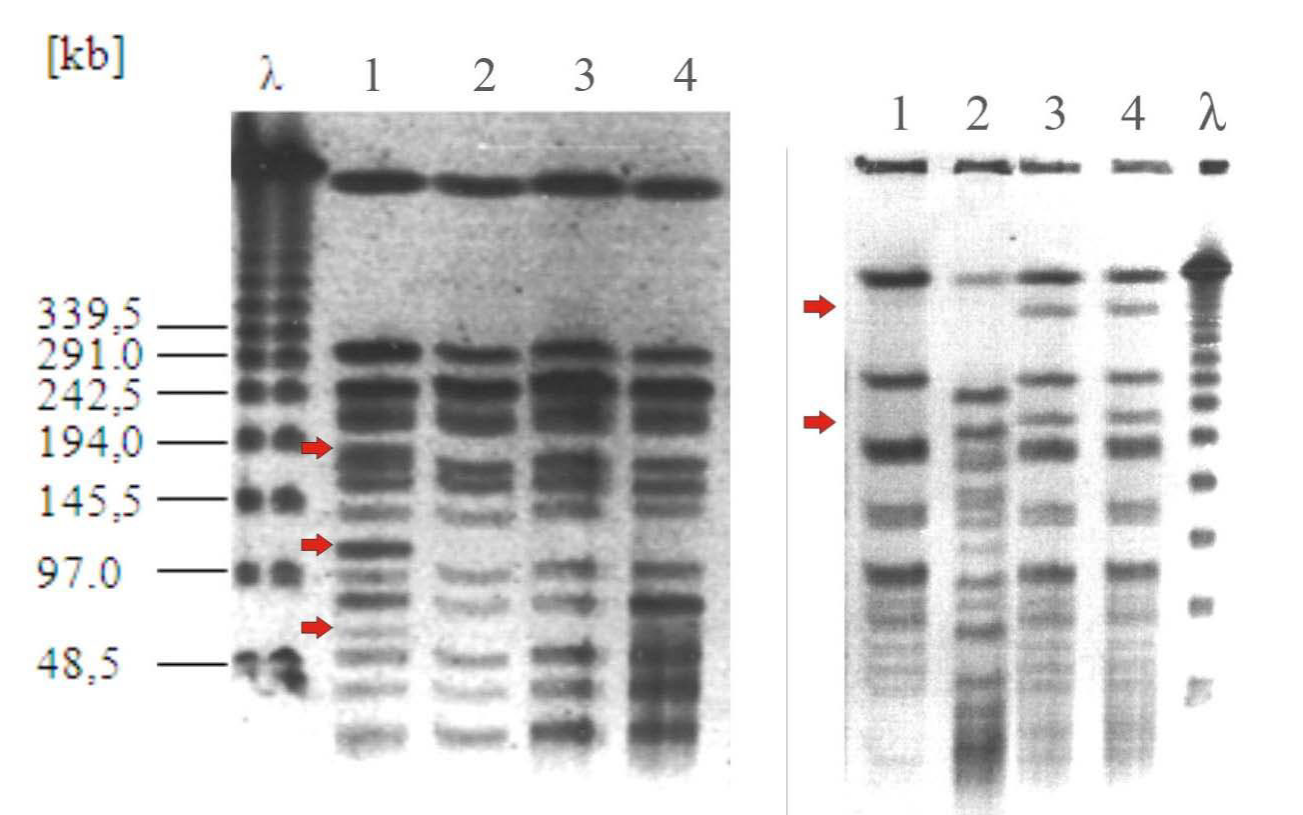
**The left panel shows genomic rearrangements of three spontaneous colony variants of *B. petrii***. Genomic DNA of *B. petrii *wild type (1), variant f (2), variant g (3) and variant k (4) was cut with *Bcu*I and separated by pulsed field electrophoresis. The red arrows indicate three bands which are missing in the three variants as compared to the wild type. The right panel shows a representative pulsed field gel of wild type *B. bronchiseptica *PS2 (lane 1), *B. petrii *(lane 2) and the two GI3::tet^R ^transconjugants of *B. bronchiseptica *(lanes 3,4) after digestion with *Bcu*I. The red arrows indicate the additional bands present in the transconjugants as compared to *B. bronchiseptica *wild type.

In a previous study we have reported about the spontaneous loss of a huge part comprising more than 500 Kb of the genome of *B. petrii *during *in vitro *culture correlating with the presence of several genomic islands (GI1–GI3) [14]. To investigate whether the frequent appearance of phenotypic variants of *B. petrii *is in fact correlated with the various genomic islands, we started to characterize the three variants described above by pulsed field gel electrophoresis. Figure 2 shows that after *Bcu*I digestion each of the three variants lack three large bands as compared to the wild type, but they have an identical restriction pattern among each other. To identify those regions of the variants lacking as compared to the wild type we performed hybridization studies with a *B. petrii *DNA-whole genome microarray. The results presented in Table 1 show that in all three variants the same genes are missing and that the deleted regions correspond to the *clc*-like elements GI1, GI3, and GI6 and to the island GI5. This shows that during *in vitro *culture these four elements have been lost in the three variants. It is surprising that all three variants exhibit identical genomic variation, since, as mentioned above, they have different growth characteristics at 15°C and colony morphology. Therefore, additional mutation(s) must have occurred which did not involve large deletions detectable by the array experiments.

**Table 1:** Characterization of spontaneous *B. petrii *variants using a DNA microarray

Predicted genomic islands (GI)	Genes present or absent in the variants g, f, and k	Presence of GI in the variants
GI (Bpet0187 – Bpet0310)	Bpet0187 – Bpet0310	**+**
GI1 (Bpet1009 – Bpet1275)	Δ Bpet1009 – Bpet1287	**-**
GI2 (Bpet1288 – Bpet1437)	Bpet1288 – Bpet1437	**+**
GI3 (Bpet1438 – Bpet1545)	Δ Bpet1438 – Bpet1545	**-**
GI4 (Bpet2166 – Bpet2216)	Bpet2166 – Bpet2216	**+**
GI5 (Bpet3699 – Bpet3770)	Δ Bpet3699 – Bpet3779	**-**
GI6 (Bpet4174 – Bpet4316)	Δ Bpet4174 – Bpet4315	**-**
GI7 (Bpet4544 – Bpet4630)	Bpet4544 – Bpet4630	**+**

The comparison of the deleted genes of the variants with those which according to the annotation are encoded on the GIs revealed a perfect congruence of the predicted island borders and the microarray data in the case of GI3, while the extent of the deletions and therefore the sizes of these elements differed from the bioinformatic prediction in the case of GI1, GI5 and GI6 [14]. According to these data, GI1 appears to comprise additional 12 genes (Bpet1267–1287), GI5 additional 9 genes (Bpet3771–3779), and GI6 appears to lack one gene (Bpet4316) (Table 1). These data were further corroborated by a series of Southern blot experiments with probes specific for the respective genes, the results of which matched perfectly with the microarray data (data not shown).

### Definition of the borders of the genomic islands of *B. petrii*

**Figure 3:**
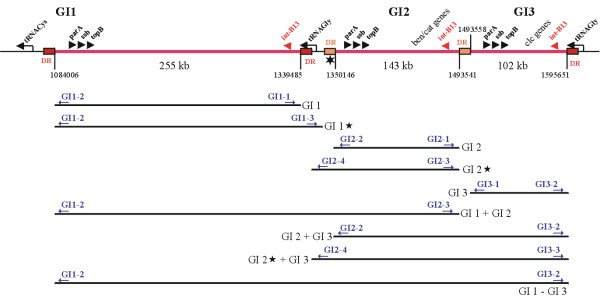
**Schematic presentation of the genomic region comprising the genomic islands GI1, GI2 and GI3**. The GIs are shown as a red lines, their flanking direct repeat regions (DR) by red boxes (dark and light red for identical or nearly identical sequences, respectively) (see also Figure 4). The sequence position of the direct repeats and the approximate size of the islands are shown below the elements. The position of tRNA genes is indicated. Some relevant or characteristic genes encoded by the islands are shown above the elements. The bars below the elements show the expected dimensions of the element after excision from the genome. Stars indicate predicted elements which may use alternative direct repeat sequences for excision or elements composed of more than one island. Arrows above the bars indicate the approximate position of PCR primers and their names (in blue) designed for the amplification of the respective circular intermediates of these elements (Tab. 3).

**Figure 4:**
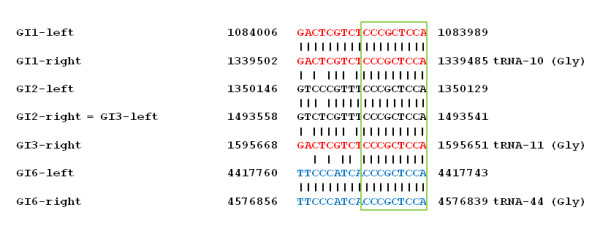
**The direct repeats generated by the integration of the *clc*-like elements in the *B. petrii *genome are shown**. Identical sequences are indicated in red or blue letters, respectively. Sequence identities are indicated by vertical bars. The positions of the sequences on the genome sequence are shown on the left and the right of the sequences. The core region identical in all repeats flanking the *clc*-like elements is indicated by the green box. In case the repeats are part of a tRNA gene, the respective gene is mentioned on the right side of the respective sequences.

Integrative and conjugative elements (ICEs) are known to be self transmissible genomic islands and their excision is mediated by the recombination between the left and right end repeats leading to a circular intermediate and the integration by the recombination between the attachment site on the chromosome (*attB*) and the conserved attachment site (*attP*) on the circular element [2,19]. To determine the exact borders of the GIs and to detect circular intermediates occurring after excision of the elements we used a PCR based approach. For the detection of excised circular intermediates of the various GIs, a series of oligonucleotide primers was designed from the presumable ends of the respective elements which are supposed to join during circularization. In the case of the adjacent elements GI1, GI2 and GI3 we considered that also various combinations might occur by common excision events of these adjacent islands (Figure 3). The direct repeats flanking the various *clc*-like elements of *B. petrii *are shown in Figure 4.

Table 2 shows the results of this analysis. In the case of GI1 no product could be amplified when using the primer pair GI1–1/GI1–2 which should provide a product, when the excision involves the direct repeat sequences directly upstream (sequence position 1,084,006) and downstream (sequence position 1,339,485) of the island. Instead, a product was obtained when the primer pair GI1–2/GI1–3 was used which can yield a product only when ring formation involved an alternative downstream repeat sequence (sequence position 1,350,146). This alternative downstream repeat sequence has three mismatches as compared to the upstream repeat and has probably been generated by the integration of GI2, since GI2 at the downstream end is flanked by a second nearly identical copy of this direct repeat (Figure 4). These data indicate that the size of GI1 is in fact larger than previously suggested and confirm the microarray data of the spontaneous variants which indicated that the genes Bpet1276–1287 are excised together with GI1 (termed GI1* in Figure 3). The genomic region comprising Bpet1276–Bpet1287 has a high GC content of about 67% which is typical for the *B. petrii *core genome. The respective genes encode hypothetical proteins, two transcriptional regulators and in addition the tRNA^Gly ^gene which was likely the original insertion point of GI1. The alternative direct repeat sequence flanking the adjacent GI2 may now be the preferred target of the GI1 integrase and may allow the element to incorporate this region of the genome thereby leading to an extension of the primordial GI1. Thus, tandem integration of genomic islands may lead to acquisition and transfer of additional genetic material of the host genome and thereby may contribute to evolution of GIs.

**Table 2:** PCR detection of excised circular intermediates of the genomic islands GI1 to GI7

	Primer combinations used	Size of the expected PCR product [bp]	PCR product obtained
GI1	GI1–1/GI1–2	1,331	**-**
GI1*	GI1–2/GI1–3	677	**+**
GI2	GI2-1/GI2–2	624	**+**
GI2*	GI2–3/GI2–4	902	**-**
GI3	GI3–1/GI3–2	967	**+**
GI1+GI2	GI2–3/GI1–2	1,175	**-**
GI2+GI3	GI3-2/GI2-2	578	**+**
GI2*+GI3	GI3-3/GI2–4	494	**-**
GI1–GI3	GI3-2/GI1–2	720	**+**
GI4	GI4-1/GI4-2	384	**+**
GI5	GI5-1/GI5-2	571	**-**
GI6	GI6-1/GI6-2	850	**+**
GI7	GI7-1/GI7-2	384	**+**

For GI2 we obtained a PCR product demonstrating the involvement of the direct repeats directly flanking the island at sequence positions 1,350,146 and 1,493,541. Since GI2 is not directly associated with a tRNA gene it appears likely that it has integrated in the left repeat of GI3 at sequence position 1,493,541, which was generated by the previous insertion of GI3 in the respective tRNA gene (tRNA-11). For GI3 we obtained the expected data which also correspond to the microarray results described above. Moreover, we obtained evidence that the *clc*-like elements GI1–GI3 can excise together in different combinations: GI2–GI3 and GI1–GI2–GI3. Therefore, these islands appear to be able to excise independently from each other, but also in various combinations thereby potentially forming composed transmissible elements. In the case of the fourth *clc*-like element, GI6, the microarray data revealed the presence of the Bpet4316 gene in the chromosome even after excision of the element. This is surprising, since the direct repeat sequence which should be the target for the GI6 integrase lies beyond this gene. Thus, the Bpet4316 gene should be located within the excised region. Curiously, the PCR experiments aiming in the detection of circular intermediates showed that the Bpet4316 gene is also part of the circular excised form of this element. This suggests a duplication of the Bpet4316 gene during excision by an unknown mechanism.

In the case of GI4 and GI7 we obtained PCR products providing evidence for excised circular intermediates which perfectly match the previous bioinformatical predictions based on the detailed sequence analysis about the size of these islands. In contrast, in case of GI5 we were not able to detect a circular intermediate neither with the originally predicted borders nor with the additional genes suggested by the microarray experiments (Bpet3771–3779), although the microarray data of the phenotypic variants f, g, and k definitely revealed the deletion of this element from their genomes.

As shown above, we were able to detect circular intermediates of most genomic islands by PCR amplification, although the microarray experiments with the phenotypic variants clearly demonstrated the deletion events. Possible explanations for this fact could be that the excised islands are diluted during growth of the bacteria since they cannot replicate. Moreover, the experimental protocols for the two methods are different and PCR amplification is much more sensitive as compared to cy3/cy5 labeling by Klenow polymerisation.

### Stability of genomic island GI3

**Figure 5:**
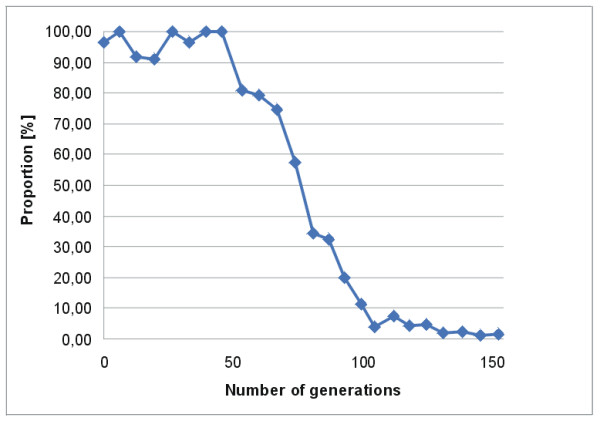
**Stability of the genomic island GI3 in the genome of *B. petrii *during culture grown without selective pressure**. On the *x*-axis the number of consecutive generations of the bacteria culture and on the *y*-axis the proportion of tetracycline resistant bacteria in the culture is shown.

The frequent appearance of phenotypic variants involving the genomic islands present in the *B. petrii *genome and the detection of circular intermediates of these islands under standard growth conditions indicates that these genomic islands are rather unstable and active at least in terms of excision. To assess the stability of one of these islands (GI3) by homologous recombination we integrated a tetracycline resistance cassette in GI3 between the genes Bpet1523 and Bpet1524 coding for a putative transposase and a glycosyltransferase, respectively. Under standard growth conditions, the resulting strain *B. petrii *GI3::tet^R ^did not show any change in its maximum specific growth rate as compared to the wild type (data not shown). This strain was then used for growth experiments without selective pressure in which the bacteria were cultivated for about 150 consecutive generations. Exponentially growing *B. petrii *has a generation time of about 90 min (data not shown). Figure 5 shows the time course of loss of GI3::tet^R ^determined by differential counting of tetracycline resistant and sensitive bacteria plated out on the respective agar plates. GI3 was stably present in the *B. petrii *population for about 40 generations, then the proportion of tetracycline resistant bacteria declined steadily and virtually no tetracycline resistant bacteria were found in the population after about 100 generations. Lack of the entire GI3 was confirmed by Southern blotting in representatives of these bacteria (data not shown). Although we cannot exclude a destabilizing effect of the tetracycline cassette on the island, it is likely that GI3 is highly unstable and gets lost with a high incidence when no selective pressure for its persistence is present.

### Transfer of genomic island GI3 to *Bordetella bronchiseptica*

**Figure 6:**
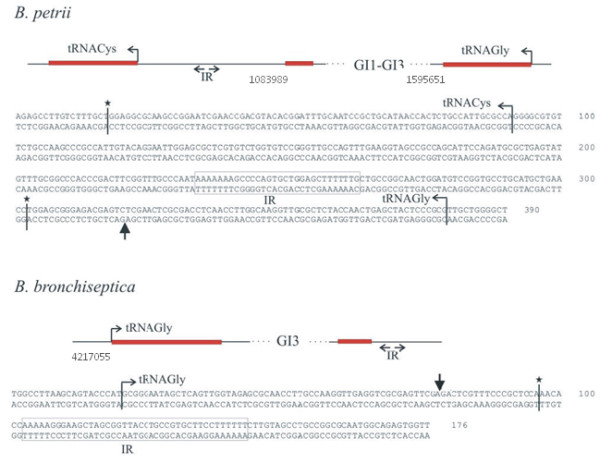
**Comparison of the integration sites of GI1–GI3 in *B. petrii *(on the top) and of GI3::tet ^R ^in *B. bronchiseptica *PS2 (below)**. Above the respective DNA sequences a schematic presentation of the integration regions is shown. In *B. petrii *GI1–GI3 is integrated in a tRNA^Gly ^gene (tRNA11) leading to an 18 bp duplication of the 3'-end of the tRNA (red boxes). On the left side of the integration side an inverted repeat (IR) is indicated. Upstream of the IR a gene encoding a tRNA^Cys ^is located. In *B. bronchiseptica *GI3::tet^R ^is once more integrated in a gene encoding a tRNA^Gly ^(tRNA45) leading to a 18 bp duplication of its 3'-end. Much alike in *B. petrii *the direct repeat sequence is followed by an inverted repeat (IR). Below the schematic presentations of the integration regions the respective DNA sequences of the integration sites are shown. The start points of the tRNA genes are indicated by horizontal arrows indicating transcriptional polarity of the genes followed by a bar marked with a star which indicates the end of the tRNA gene. Vertical arrows indicate the integration sites of the GIs in the tRNA genes. Related inverted repeat sequences (IR) present in both species are boxed. In the case of *B. bronchiseptica *the sequence position indicated is taken from the genome sequence of strain RB50 [13].

In the case of the ICE*clc *self transmission to a variety of β- and γ-*Proteobacteria *was observed [20]. Since the *clc*-like element GI3 is active at least in terms of excision from the chromosome, we investigated its capacity to transfer itself to another host. Therefore, the above described *B. petrii *GI3::tet^R ^strain carrying a tetracycline resistance gene in GI3 was used for conjugation experiments with *B. bronchiseptica*. As a recipient *B. bronchiseptica *PS2 was used which carries a Tn*phoA *insertion in the genome conferring kanamycin resistance [21]. Transconjugants were selected by their resistance against kanamycin and tetracycline. Two transconjugants were isolated and further characterized by pulsed field gel electrophoresis after restriction of the genomic DNA with *Bcu*I. Both strains showed two additional bands of the same size, which is in agreement with the fact that the only *Bcu*I restriction site in GI3::tet^R ^is located in the tetracycline gene cassette (Figure 2). To identify the integration site of GI3::tet^R ^in PS2 we used a PCR based approach. Since *clc*-like elements are known to preferentially integrate in genes coding for tRNA^Gly ^we designed oligonucleotides to amplify the four tRNA^Gly ^genes present in *B. bronchiseptica*. For three out of the four tRNA genes we obtained PCR products of the expected size. Only in the case of the BBt45 gene no PCR product was obtained suggesting the integration of GI3::tet^R ^in this tRNA gene (data not shown). To identifiy the exact insertion site we used primers GI3-2 and GI3-1 from the two ends of GI3::tet^R ^and designed additional primers (tRNA45-1 and tRNA45-2) from the neighbouring sequences of the BBt45 gene. As expected, using the primer pairs GI3-2/tRNA45-1 and GI3-1/tRNA45-2 we obtained two PCR products of 625 bp and 647 bp, respectively. Sequence analysis of these products confirms the integration of GI3::tet^R ^in the BBt45 gene and reveals the duplication of the last 18 bp of the tRNA^Gly ^gene and an inverted repeat sequence in the direct neighbourhood. The duplicated sequence is identical with the direct repeats in *B. petrii *flanking GI1 on both sides and GI3 on the right side (Figure 6). Similarily, the inverted repeat sequence in the proximity of the integration site in *B. bronchiseptica *resembles inverted repeat sequences associated with the integration sites of ICE-GI3 of *B. petrii *and ICE*clc *in *Pseudomonas knackmussii *sp. strain B13 [22]. The fact that GI3 can actively excise and reintegrate into the genome of a recipient strain proves this island to be a functional integrative and conjugative element and therefore it should be renamed ICE-GI3.

## Conclusion

The data presented here underline the previous notion of a highly mosaic genome of *B. petrii*. By microarray analysis of spontaneous phenotypic variants of *B. petrii *and by direct detection of excised circular intermediates of the *B. petrii *GIs we show that all of them are active at least in terms of excision. We provide evidence that the adjacent integration of highly related elements may enable these elements to pick up additional genomic material placed between the integration sites thereby leading to an increase in the size of the islands. Moreover, the adjacent placement of islands encoding highly similar integrases and attachment sites may also lead to the formation of novel huge composite islands. For ICE-GI3 we show that without selective pressure this island is lost from the bacterial population. Moreover, we show that this island is self transmissible and can be transferred to another *Bordetella *species, *B. bronchiseptica*. Therefore, the evolution of *B. petrii *involved massive horiztonal gene transfer, while in the classical pathogenic *Bordetella *species only very few examples of HGT have been reported, e.g. the horizontal transfer of insertion elements, the acquisition of an genomic region encoding an iron uptake system in *B. holmesii *and, possibly, the inactivation of the genes encoding adenylate cyclase toxin in a specific *B. bronchiseptica *lineage by a horizontally acquired gene cluster encoding peptide transport genes [12,23,24]. This may indicate that their unique habitat due to an obligate host association has dramatically limited the impact on horizontal gene transfer for the pathogenic Bordetellae once they had acquired their capacity to infect and to persist exclusively in vertebrate hosts.

## Methods

### Bacterial strains and growth conditions

In this study *B. petrii *DSM12804, the type strain of the species [5], *B. bronchiseptica *BB7866 [25], and *B. bronchiseptica *PS2, carrying a Tn*phoA *insertion in the genome, conferring kanamycine resistance [21], were used. *B. petrii *was routinely grown in LB broth, while *B. bronchiseptica *strains were cultured on BG-agar plates or in SS-liquid medium, as previously described [26]. If necessary, antibiotics were added to the culture media in the following concentrations: tetracycline, 12.5 μg/ml; kanamycine, 50 μg/ml. Conjugation experiments were carried out by filter mating as described previously [26]. The long time survival experiments were carried out as described by Preston and Wardlaw [18]. For this purpose sterile filtered river water from the river Main was inoculated with bacteria *B. bronchiseptica *BB7866 and *B. petrii *(2,000 CFU/ml), respectively, and incubated at 37°C. Samples were taken at different time intervals up to 263 days after inoculation and bacterial number was counted by plating out serial dilutions of the bacteria.

### Molecular genetic tools

DNA manipulations including cloning, restriction analysis, DNA-sequence analysis, preparation of genomic DNA, Southern blots were carried out according to standard procedures. In all cases, chromosomal DNA used for PCR reactions or for whole genome hybridization analyses was purified from bacterial cultures inoculated from single colonies on agar plates. Pulsed field electrophoresis was carried out with the BioRad CHEF-DRII system as described previously [5].

### *B. petrii *DNA microarray specifications and hybridization conditions

Sixty-mer oligonucleotides sequences were designed as described previously using OligoArray2.0[27]. Lyophilised 5'-aminated oligonucleotides (Sigma Aldrich) were then resuspended in SciSPOT AM 1× buffer at 20 μM final concentration before being spotted on aldehyde coated Nexterion slides AL (Schott) using QArray2 (Genetix) spotter. Slides were then incubated at room temperature in a humidity chamber (> 90% relative humidity) and then in an oven at 120°C during 1 hour. Slide surface was then blocked twice for 2 min in 0.2% SDS solution, then twice for 2 min in RNase-DNase free water. The slides were then incubated at room temperature during 15 min in 125 mM NaBH_4 _prepared extemporally in a 3:1 (vol/vol) PBS:Ethanol mixture. The slides were then rinsed twice for 2 min in 0.2% SDS, then twice for 2 min in RNase-DNase free water and dried before hybridisation.

### Genomic DNA extraction and labelling

Genomic DNA used for the microarray experiments was prepared by using the Genomic-tip 100/G anion exchange columns (Qiagen), following the manufactor's recommendation. 20 μg of the genomic DNA was digested with *MboI *restriction enzyme (2 U/μg, Fermentas) at 37°C for 2 hours and complete restriction was confirmed by agarose gel electrophoresis. The fragmented genomic DNA was purified with phenol-chloroform-isoamyl alcohol (25:24:1). Aliqouts of 2 μg of genomic DNA were labelled using the Amersham Nick Translation Kit N5500 (GE Healthcare) in the presence of 91.3 μM dATP, 91.3 μM dGTP, 91.3 μM dTTP, 26.1 μM dCTP, and 33 μM Cy3-dCTP or Cy5-dCTP. Cy-labelled dCTP was obtained from Perkin-Elmer. After incubation at 15°C in the dark for 4 hours, the labelled genomic DNA was purified using the QIAquick Spin PCR Purification Kit (Qiagen).

### Microarray hybridisation and data analysis

Genomic DNA from the reference strain *B. petrii *DSM 12804 was hybridised against each *B. petrii *variant (g, k, f) in a dye-swap experimental design. Labeled genomic DNA was resuspended in 480 μl of hybridisation buffer containing 40% deionised formamide, 5× Denhardt's solution, 50 mM Tris pH 7.4, 0.1% SDS, 1 mM Na pyrophosphate, and 5× SSC, denatured at 95°C for 3 min and hybridised to the *B. petrii *microarray for at least 12 hours at 52°C. After hybridisation the microarrays were washed for 5–8 min at 42°C with wash buffer (2× SSC, 0.2% SDS), in 0.5× SSC for 10 min and in 0.05× SSC for 5 min at room temperature. A last rinse was carried out in 0.01× SSC for 30 sec before the microarrays were dried by centrifugation for 5 min at 200 g. The arrays were scanned using an Innoscan 700 (Innopsys) microarray scanner, and analyzed with ImaGene 8.0.0 (BioDiscovery). Normalisation of the data was carried out with R Project for Statistical Computing http://www.r-project.org. The following genome typing analysis was performed with the program GACK http://falkow.stanford.edu/whatwedo/software.

### Determination of circular intermediates of the genomic islands by PCR

To detect circular intermediates in the case of the *B. petrii *islands oligonucleotides were designed such that in PCR reactions amplification products can only be obtained when the elements are circularised. The PCR primers used for the detection of circular intermediates of the various genomic islands are shown in Table 3. The expected products of these PCR reactions are listed in Table 2. In case of successful amplification the PCR products were sequenced to confirm the specificity of the amplification.

**Table 3:** Oligonucleotides used in this study

Designation	DNA-Sequence
GI1-1	5'-TAC GGA CCT TCT CGG CGG-3'
GI1–2	5'-GAC CCA AGG CAA GAC GCT G-3'
GI1–3	5'-ATT ACC CGC ATT CCC TTG TTG-3'
GI2-1	5'-TCG TTG ACC TCG CTC CTC CA-3'
GI2-2	5'-TAC GAC AGT TGA CCA CAG TTG-3'
GI2–3	5'-CTC TGC CGT CCC TCC TTG-3'
GI2–4	5'-TCA AGA CCA TCG TAT AGC GG-3'
GI3-1	5'-AGG TCT AGG AAA ACT GGG CGA ATC-3'
GI3-2	5'-GTA TTC CTG TGC CTA GAT TGG-3'
GI3–3	5'-TCA GCC CCA GCA ACT ATC C-3'
GI4-1	5'-ATG AAC ACC CGG CGA CCC-3'
GI4-2	5'-GAG CTA ACC TAC TGT CCC AT-3'
GI5-1	5'-GTT TTG GGA TGT TTT GAA GCG TG-3'
GI5-2	5'-CGG TCG AAG AAG CCA GCA GT-3'
GI6-2	5'-GAT AGG GTT CGC TCA CAC GGC-3'
GI6-1	5'-CTC CTC CAG CAA CAA TAC GG-3'
GI7-1	5'-TTG AGA CGA CTA TGA ACC CAG-3'
GI7-2	5'-CGC CCA TTG CCA CGA CCG-3'
Tet1	5'-GAC GGC GGC CGC ATC TGG CAA AGC-3'
Tet2	5'-ATA CTA GTC ATC GCG TGA TCC TCG CGA A-3'
Tet3	5'-ATG AAT TCA ATA CGC CCG AGA CCC GCG-3'
Tet4	5'-CAT CTC GAG AAA ACG GTG AAG GCC AGC-3'
tRNA45-1	5'-CCG TCT CCA ATC CCA AGG C-3'
tRNA45-2	5'-CTG GAA CAA GAA GGC CG C-3'

### Construction of a *B. petrii *strain harbouring a tetracycline resistance gene in GI3

The insertion of a tetracycline resistance cassette in the genomic island GI3 was performed by homologous recombination using a tetracycline resistance cassette derived from the cloning vector pBR322 flanked by *B. petrii *derived sequences. Briefly, for this purpose two DNA fragments derived from the intergenic region of the *B. petrii *genes Bpet1523 and Bpet1524 encoded on GI3 were amplified using the PCR primer pairs Tet1/Tet2 and Tet3/Tet4 (Table 3) which harboured restriction sites for *Not*I and *Bcu*I (Tet1/Tet2) and for *Eco*RI and *Xho*I (Tet3/Tet4), thereby providing suitable ends for ligation with the tetracycline gene cassette. The tetracycline gene was ligated with the amplified DNA fragments and cloned into pBluescript KS cut with *Not*I and *Xho*I. The plasmid harbouring the tetracycline cassette was then purified and electroporated into *B. petrii *according to standard procedures using a Micropulser (BioRad, Germany). Bacteria were then plated on LB agar plates containing tetracycline to select for integration of the tetracycline cassette into the genome. Resulting clones were checked by Southern blotting and PCR analysis for proper integration of the resistance cassette at the desired position on GI3. The resulting strain *B. petrii *GI3::tet^R ^was used for conjugation experiments and for the analysis of island stability. These experiments were carried out as described previously [28]. Briefly, overnight cultures (15 h, 37°C) of the strain were diluted 1:100 in 30 ml of LB broth. Bacteria were incubated at 37°C and samples were taken during the late lag, mid-log, early stationary, and late stationary phases. The identification of spontaneously arising tetracycline sensitive clones was performed by plating out serial dilutions on LB agar plates with and without tetracycline.

## Authors' contributions

ML performed most of the experimental work. KS performed the DNA-microarray experiments. SB performed cloning and conjugation experiments. DH, CH, EL and YL developed and validated the *B. petrii *DNA microarray. CL and YL coordinated the development and validation of the DNA microarray. RG coordinated the work, designed the experiments and drafted the manuscript. All authors read and approved the manuscript.
